# Pharmacological and Non-pharmacological Treatments of Irritable Bowel Syndrome and Their Impact on the Quality of Life: A Literature Review

**DOI:** 10.7759/cureus.9324

**Published:** 2020-07-21

**Authors:** Reham Gendi, Nusrat Jahan

**Affiliations:** 1 Internal Medicine, California Institute of Behavioral Neurosciences & Psychology, Fairfield, USA

**Keywords:** irritable bowel syndrome, physiology, pathology, epidemiology, psychology, therapy

## Abstract

Irritable bowel syndrome (IBS) is a non-organic gastrointestinal disorder that adversely affects the quality of life (QoL). The etiology of the disease is not fully understood, and appropriate treatment is still a matter of debate. Therefore, we feel that a review that describes the different treatment methods of treatment is needed to provide the correct clues. A comprehensive literature search was performed on PubMed, including randomized clinical trials and review articles. We carefully reviewed the different treatment options mentioned in the published research papers and compared their results to address the treatment options for IBS. The current literature review reveals that pharmacological treatments such as antidepressants, vitamin D, probiotics, and antispasmodic drugs and non-pharmacological treatments including cognitive-behavioral therapy (CBT), acupuncture, anise, and diet modification can help control the symptoms of IBS and improve the QoL of patients.

## Introduction and background

Irritable bowel syndrome (IBS) is a non-organic gastrointestinal disorder that has a negative impact on the quality of life (QoL). Currently, the diagnosis of IBS is based on new Rome IV criteria as a recurring abdominal pain related to two or more of the following conditions: defecation-related, associated with an increase in the stool frequency, and associated with a disparity in stool quality [1]. Symptom onset should occur at least six months before the diagnosis, and symptoms should be presented in the last three months, on average, and at least one day in a week.

The prevalence of IBS is approximately 9-23% worldwide, with a devastating impact on the QoL. Hence, we wanted to find out if IBS could be associated with psychological problems. The currently available treatment options, both pharmacological and non-pharmacological, can help in controlling symptoms. However, a knowledge gap still exists. We aim to find the best possible treatment options for IBS, which would help to treat this condition and improve the QoL for patients.

Mechanism of IBS

IBS symptoms have unclear etiology, and there is no specific treatment available to treat IBS patients. Recent studies show that symptoms of IBS most likely occur due to the gut-brain axis imbalance as evidenced by increased abdominal pain during periods of stress. Stress can cause a change in intestinal motility and permeability along with microbiota imbalance as some cases developed IBS due to alteration of the gut microbiota as a result of an infection [1,2-10]. In contrast, some believe it to be mostly caused by psychological disorders. According to Spiller et al., at least 50% of the IBS patients can be described as depressed, anxious, or hypochondriacs and over 60% of IBS patients receiving tertiary care had presented with a psychiatric disorder, most commonly depression, anxiety, or both [11].

Psychological disorders and their relation to IBS

Stress can modify and exacerbate central pain circuity and, therefore, it may be related to the development of visceral pain, which is related to IBS. A study has shown that IBS could be related to mental disorders as about 50-90% of IBS patients meet the criteria of psychiatric disorder, mainly anxiety, panic disorder, and major depressive disorder [12]. Another study has shown that catastrophizing, which is defined as expecting a negative outcome, is more common in IBS patients [13]. Hence, IBS can be associated with pain-catastrophizing thinking with a propensity to magnify the seriousness of the illness.

Treatment options

We are currently aware of the multi-systemic reasons behind the occurrence of IBS symptoms and the role of psychological disorders in its development or exacerbation. However, it needs to be further established as to how psychological treatment plans can be used as an effective way to resolve IBS symptoms. This psychosomatic visceral pain link can help us to illustrate several treatments that can help reduce symptoms in patients with IBS and improve their QoL.

The objective of this review is to focus on the treatment options for IBS and the effect of using different interventions to control IBS symptoms. It compares various studies discussing cognitive behavioral therapy (CBT), antidepressants, probiotics, anise, acupuncture, vitamin D, and diet modification as management options for IBS. The literature review aims to provide an insight into current treatment options for controlling IBS symptoms and improving the QoL.

## Review

Materials and methods: data collection

The data were collected from PubMed using Medical Subject Headings (MeSH) keywords “Irritable bowel syndrome, physiology, pathology, epidemiology, psychology, and therapy.” The following inclusion criteria were applied in the order given; clinical trial and review studies, articles involving only human subjects, articles published in English only, and those involving subjects aged 19-44 years. The collection of the articles for the study was done in an ethical manner.

Results

Using MeSH keywords on PubMed, a total of 5,780 papers were collected, of which 651 were used for filtering. The details of the articles obtained by applying MeSH keywords and inclusion criteria in PubMed are given below (Table 1).

**Table 1:** Article search results on PubMed MeSH: Medical Subject Headings

Articles	Results, n
Total article found on PubMed	13,950
Articles obtained with MeSH keywords	5,780
Clinical trials and reviews	2,221
Human subjects	2,215
English language	1,976
Subjects aged 19-44	651

Each of the 651 studies identified was evaluated individually based on the abstract. Among them, 163 articles were nonduplicated, and 67 were found to be non-relevant while screening the abstracts. Another 12 articles were excluded after the full-text screening since the patient age was out of the age range for our study. And one more article was removed during data extraction as it was a systemic review that included different age groups.

Finally, 83 relevant articles were chosen for this review article. The current literature review analyzes a total of 16 full-text articles that study different treatment options for controlling IBS symptoms.

Discussion

Although IBS is not a life-threatening condition, it puts a heavy burden on healthcare resources. Treating a patient for one year costs around $355-3,344, and the condition has a very drastic effect on the QoL of patients [14]. The condition has an unknown mechanism, and it has been found to alter intestinal motility and cause stress and microbiota imbalance, which explains the treatment options available now [1,2-10]. In this review, we included 83 papers on PubMed, which addressed the various treatment options available for IBS. We also engage in a comparison of these studies.

It has been found that many IBS patients respond to treatment to some extent. However, others do not due to various factors or the disparity in severity. In light of this, we review both pharmacological and non-pharmacological treatments that aimed to provide the best control of IBS symptoms and their effects on QoL. Our review aims to provide a complete overview of these treatment options to determine their efficacy in treating IBS in light of these previous studies.

Effect of Cognitive Behavioral Therapy on IBS

One study has discussed cognitive behavioral therapy (CBT) as a treatment option for controlling IBS symptoms compared with other possibilities as an educational exercise [15]. Another one has discussed its mechanism by drawing a connection between emotion, behavior, and cognition as a treatment option for IBS [16]. Overall, all these studies generally endorse using CBT for IBS (Table 2).

**Table 2:** Studies that discuss the effect of CBT on IBS CBT: cognitive behavioral therapy; IBS: irritable bowel syndrome; QoL: quality of life

Study	Author name/year	Study design	Sample size	Main points
Study 1	Lackner et al./2018 [15]	Randomized controlled trial	436 patients	The study compared CBT vs. education in the treatment of IBS. Patients were more responsive to CBT than education for IBS symptoms control
Study 2	Reme et al./2011 [16]	Randomized controlled trial	149 patients	This study supports that CBT has a positive impact on IBS symptoms due to communication between emotion, behavior, and cognition in the development of IBS
Study 3	Jang et al./2014 [17]	Randomized controlled trial	180 patients initially; 13 patients withdrew during the research for various reasons	The study showed the effect of CBT on IBS in female nursing students. CBT proved to be effective in improving QoL and reduced the symptoms of IBS
Study 4	Chilcot et al./2013 [18]	Randomized controlled trial	64 patients	The study showed CBT has a better effect on symptoms of IBS rather than usual treatment. CBT has a positive effect on IBS due to its impact on cognition rather than the mood
Study 5	Ljótsson et al./2013 [19]	Randomized controlled trial	195 patients	This study compared the impact of internet CBT and stress management on improving IBS symptoms. Internet CBT has a positive effect on IBS symptoms due to its ability to reduce gastrointestinal anxiety and not due to stress reduction
Study 6	Ljótsson et al./2010 [20]	Randomized controlled trial	85 patients	This study examined the role of CBT in developing mindfulness and exposure offered by the internet in controlling IBS symptoms. It showed that CBT via the internet is beneficial for controlling IBS symptom and improving patients’ QoL

Effect of Anti-psychological Pharmacological Treatment on IBS

Antidepressants and antipsychotic medications can be a treatment option for IBS. Many of the studies illustrate that some of these drugs, such as duloxetine, have shown a positive effect on patients with generalized anxiety disorder (GAD) along with IBS [21]. Tianeptine and amitriptyline have a positive impact on irritable bowel syndrome with diarrhea (IBS-D) [22]. Imipramine can also be used to treat IBS [23]. One study showed that citalopram could be useful while another study questioned this conclusion; both studies discussed the effect of citalopram on IBS symptoms away from its impact as an antidepressant (Table 3).

**Table 3:** Studies that discuss the effect of pharmacological treatment on IBS IBS: irritable bowel syndrome; IBS-D: irritable bowel syndrome with diarrhea; GAD: generalized anxiety disorder

Study	Author name/year	Study design	Sample size	Main points
Study 1	Kaplan et al./2013 [21]	Pilot study	13 patients	This study analyzes the effect of duloxetine in 13 patients with GAD and IBS. It found that duloxetine has a positive impact on IBS and GAD symptoms
Study 2	Sohn et al./2012 [22]	Randomized clinical trial	228 patients	This study compared the effect of tianeptine with amitriptyline on IBS. It used both medications in addition to probiotic. it concluded that both can improve IBS-D, but the side effects of tianeptine limit its use
Study 3	Abdul-Baki et al./2009 [23]	Randomized, double-blind trial	56 patients	Imipramine has a positive impact on IBS symptoms compared to placebo. It assists in improving QoL. Precaution by choosing the right candidate for its use is essential along with starting with low dose
Study 4	Ladabaum et al./2010 [24]	Randomized clinical trial	54 patients	The research concluded that citalopram does not have a positive effect on IBS symptoms in comparison to placebo in non-depressed patients
Study 5	Masand et al./2009 [25]	Double-blind, randomized controlled study	72 patients	Paroxetine has no beneficial effect on pain control in IBS, while it has a positive impact on clinical global impression improvement ratings
Study 6	Talley et al./2008 [26]	Randomized controlled study	51 patients	Neither imipramine nor citalopram has a positive effect on global IBS endpoints
Study 7	Tack et al./2006 [27]	Double-blind, parallel-arm study	23 patients	Citalopram has a positive impact on IBS symptoms. Citalopram’s positive effect is not related to improving depression or anxiety symptoms

Effect of Probiotics on IBS

One study has shown that probiotics could improve IBS symptoms, while another one has indicated that it might aggravate the IBS symptoms, specifically bloating [28,29]. We reviewed six studies relating to probiotics, and three of them showed that the probiotic effect is no stronger than that of placebo; one study showed that the probiotic effect on improving QoL is related to its impact on depression symptoms and not on IBS symptoms [30]. On the other hand, the other three showed that probiotics do have a positive effect on controlling IBS symptoms (Table 4).

**Table 4:** Studies that discuss the effect of probiotics on IBS IBS: irritable bowel syndrome; IBS-D: irritable bowel syndrome with diarrhea; QoL: quality of life

Study	Author name/year	Study design	Sample size	Main points
Study 1	Ishaque et al./2018 [28]	Double-blind trial	400 IBS-D patients	Probiotic has a beneficial effect on IBS-D symptoms, including abdominal pain and frequency of stool
Study 2	Pinto-Sanchez et al./2017 [30]	Randomized, double-blind controlled study	44 IBS-D or mixed stool pattern patients	Probiotic Bifidobacterium longum improves depression symptoms associated with IBS and QoL, which was accompanied by a change in a brain-stimulating pattern but does not affect IBS symptoms or the anxiety related to it
Study 3	Hod et al./2017 [31]	Double-blind, placebo-controlled study	107 patients	Probiotic BIO 25 has the same effect on IBS-D symptoms as a placebo
Study 4	Han et al./2016 [32]	Randomized, double-blind controlled study	50 IBS-D patients	The research compared the effect of a double coating or non-coating probiotic. It showed that double coating probiotic relieved IBS inconvenience and can be used as a treatment option for IBS-D
Study 5	Begtrup et al./2013 [33]	Randomized controlled trial	131 patients	The research compared the effect of probiotic-containing lactobacillus Paracasei, acidophilus and Bifidobacterium Bb12 to that of placebo through six months on controlling symptoms of IBS. The study did not detect a positive effect of probiotic when compared with placebo
Study 6	Enck et al./2009 [34]	Double-blind controlled trial	298 patients	The study compared the effects of probiotic Symbioflor-2 (E-Coli product) with that of placebo on abdominal pain score and showed that probiotic has a more significant effect than placebo

Effect of Other Treatment Options on IBS

There are plenty of other treatment options that might improve IBS symptoms. These options include a low fermentable oligo-, di-, mono-saccharides, and polyols (FODMAP) diet, anise, vitamin D, acupuncture, and antispasmodic drugs [35-39]. The following table provides the details of the studies that examined the effects of each of these options on controlling IBS symptoms (Table 5).

**Table 5:** Studies that discuss the effect of low FODMAP diet, anise, vitamin D, acupuncture, and antispasmodic drugs on IBS FODMAP: fermentable oligo-, di-, mono-saccharides, and polyols; IBS: irritable bowel syndrome; IBS-D: irritable bowel syndrome with diarrhea; NICE: National Institute for Health and Care Excellence

Study	Author name/year	Study design	Sample size	Main points
Study 1: FODMAP diet effect	Eswaran et al./2016 [35]	Randomized controlled trial	84 IBS-D patients	The study compared the use of low FODMAP diet to modified NICE diet in controlling IBS symptoms and illustrated that low FODMAP diet has a significant effect on controlling pain and bloating compared to a diet based on the modified NICE guidelines
Study 2: anise effect	Mosaffa-Jahromi et al./2016 [36]	Three-armed, double-blind clinical trial	120 patients	The study illustrated that the anise cap has a better impact on IBS symptoms improvement compared to Colpermin^TM^ or placebo
Study 3: vitamin effect	Abbasnezhad et al./2016 [37]	Double-blind, randomized, placebo-controlled trial	90 patients	The study evaluated the effect of vitamin D compared to placebo on controlling IBS symptoms over six months. It showed that vitamin D has a more significant positive impact on improving IBS symptoms
Study 4: acupuncture effect	MacPherson et al./2012 [38]	Two-arm, pragmatic, randomized controlled study	233 patients	The study compared acupuncture with usual care used in controlling IBS symptoms and illustrated that acupuncture has better results and should be a treatment option for IBS
Study 5: antispasmodic effect	Zheng et al./2015 [39]	Prospective, double-blind, placebo-controlled trial	427 patients in three hospitals in China	Pinaverium has a better effect on improving IBS abdominal pain and stool frequency and can be considered a first-line treatment for IBS

Limitation of the study

Our literature review has some limitations. The study is limited in its analysis in terms of patient age (all patients involved were <45 years old) and does not include observational and non-randomized clinical trials. More studies are needed to explore the primary treatment of IBS to minimize its effects on QoL and reduce the burden on medical resources.

## Conclusions

This literature review discussed the pharmacological and non-pharmacological treatment strategies for IBS based on previous studies. Based on our findings, we can conclude that the use of methods including CBT, anti-psychological medications, probiotics, and other approaches such as acupuncture, anise, and diet modifications can help control the symptoms of IBS and improve the QOL of patients. 
